# Radiological Evolution of Fat Graft Used for Optic Neuropexy During Surgery for Parasellar Meningiomas

**DOI:** 10.1227/neu.0000000000002351

**Published:** 2023-01-20

**Authors:** Simon Diaz, Daniele Starnoni, Constantin Tuleasca, Vincent Dunet, David Peters, Mahmoud Messerer, Marc Levivier, Roy Thomas Daniel

**Affiliations:** ‡ Department of Neurosurgery, University Hospital of Lausanne and Faculty of Biology and Medicine, University of Lausanne, Lausanne, Switzerland;; § Signal Processing Laboratory (LTS 5), Ecole Polytechnique Fédérale de Lausanne (EPFL) Lausanne, Lausanne, Switzerland;; ‖ Department of Medical Radiology, University Hospital of Lausanne and Faculty of Biology and Medicine, University of Lausanne, Lausanne, Switzerland

**Keywords:** Meni, ngioma, Parasellar, Radiosurgery, Gamma Knife, Chiasmopexy, Fat graft

## Abstract

Stereotactic radiosurgery (SRS) is an effective adjuvant therapy for residual tumor after subtotal resection of parasellar meningiomas. Fat graft placement between the optic nerve/chiasm and residual tumor (optic neuropexy [OPN]) allows for safe SRS therapy.

**OBJECTIVE:**

To evaluate the radiological temporal profile of the fat graft after OPN, immediately after surgery and at 3, 6, and 12 months intervals, to elucidate the optimal time point of adjuvant SRS.

**METHODS:**

A single-center, retrospective, cohort study of 23 patients after surgery for parasellar meningioma was conducted. Fat graft volume and MRI signal ratios were calculated. SRS dosimetric parameters (tumor/optic nerve) were measured at the time of SRS and compared with a hypothetical dosimetric plan based on an early postoperative MRI.

**RESULTS:**

Of 23 patients, 6 (26%) had gross total resection and 17 (74%) had subtotal resection. Fat grafts showed a progressive loss of volume and signal ratio over time. Radiosurgery was performed in 14 (82.3%; 8 hypofractionated radiosurgery and 6 single fraction). At 3 months, there is a loss of 46% of the fat volume and degradation of its tissue intensity, decreasing differentiation from tumor and nerve. The hypothetical treatment plan (performed on an early postoperative MRI) showed that single-fraction SRS would have been possible in 6 of the 8 hypofractionated cases.

**CONCLUSION:**

OPN is a technique that can be safely performed after resection of parasellar meningiomas. Because of the reduction of the fat volume and tissue differentiation between fat and tumor/nerves, adjuvant radiosurgery is better performed within the first 3 months after surgery.

## Graphical abstract

**Figure:**
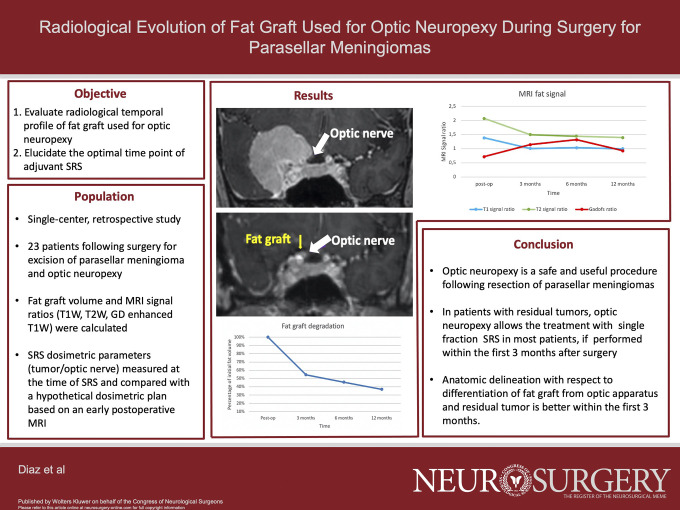

**ABBREVIATIONS:**
CS: cavernous sinus
FU: follow-up
GK: Gamma Knife
Gy: gray
GTR: gross total resection
hfRS: hypofractionated radiosurgery
OPN: optic neuropexy
RION: radiation-induced optic neuropathy
SRS: stereotactic radiosurgery
WHO: World Health Organization.

Meningiomas arising from the parasellar region represent 15% of all meningiomas and remain a challenging pathology because of their intimate relationship to vital neurovascular structures.^1^ As a result, gross total resection (GTR) is only achieved in 11.8% of patients. Current recommendations advise against microsurgical resection of cavernous sinus (CS) involvement if resection cannot be performed without compromising cranial nerve function.^1^ Main factors limiting the extent of resection are involvement of the CS, adventitia of large vessels, or optic foraminal dural.^1^ Stereotactic radiosurgery (SRS) is safe and effective for treating CS tumors, with local control up to 85%^2^ and 80% to 100% neurological preservation rates.^2^ Although CS structures are considered radioresistant, the optic nerve is considered radiosensitive and should not receive more than 12 Gy,^1,3-5^ to avoid radiation-induced optic neuropathy (RION).^6^ The other alternative is to use hypofractionated radiosurgery (hfRS), whenever tumor is in contact with the optic apparatus, and single fraction is not safely feasible.^7^

A technical solution to meet these challenges is to place a fat graft between the optic nerve and residual tumor (optic neuropexy, OPN). This maintains the distance gained at surgery and further allows an optimal treatment dose while keeping nerve exposure below 8 to 10 Gy.^1,3-5^ This technique has been previously reported to increase distance between normal pituitary gland and residual tumor after subtotal resection of pituitary tumors, facilitating adjuvant treatment with SRS/radiotherapy and effectively reducing the incidence of radiation-induced hypopituitarism.^3-5^

However, no current consensus exists on the advantages of this technique for the treatment of parasellar meningiomas. Limited data are available regarding the volumetric and radiological signal profile of the fat graft over time, which may have direct implications on SRS methods, timing of treatment, and interpretation of radiological images necessary for treatment planning.

We aimed to evaluate the temporal profile of the radiological evolution of the fat graft after OPN and its implication on postoperative SRS planning. We also aimed to identify the correct timing of adjuvant SRS by analyzing the effectiveness of fat in maintaining the distance gained at surgery.

## METHODS

We performed a single-center, retrospective, consecutive cohort study of patients treated between 2010 and 2020, in whom the OPN technique was used after resection of a parasellar meningioma. Institutional board review approval and written informed patient consent was obtained. Meningiomas were classified into clinoidal, tuberculum sellae, or spheno-orbital. Patients with a previous surgery or with less than 1-year follow-up were excluded. All patients underwent preoperative computed tomography and MRI to determine tumor characteristics such as CS extension, vessel encasement, and relationship to optic apparatus. Extent of resection was evaluated based on Simpson^8^ grading. Postoperative MRI was performed within 48 hours and then at 3-month, 6-month and 12-month follow-up. Images were reviewed by a neuroradiologist to determine the volume and to calculate the ratio of intensity of different sequences. The definition of GTR was defined as macroscopically complete resection confirmed by 3-month postoperative MRI. Fat graft was delineated at each time point (volume in mm^3^).

Examinations were performed on different 1.5 (8.8%) or 3 (91.2%) T MRI (Siemens; 3-mm thick unenhanced T1, T2). After injection of Dotarem (Gerbet), 7.4% of examinations included 3-mm thick T1 fat sat (before 2013), 83.8% had 0.9 mm thick isotropic 3D T1 fat sat spin echo sequence, and 8.8% did not include a fat sat sequence after contrast media injection. Fat graft was defined as tissue appearing hyperintense both on unenhanced T1 and T2 near optic nerve while glue appeared hypointense on unenhanced T1 and hyperintense on T2 without enhancement. Residual tumor appeared hypointense on unenhanced T1 and isointense to hypointense on T2 with enhancement. Fat graft signal was assessed on all magnetic resonance sequences using a 5-mm^2^ ellipsoid region-of-interest on its epicenter and a alike region-of-interest on the pons to calculate the signal ratio (signal of the graft that of pons). The signal ratio was favored to absolute value to account for interscanner and interpatient variability. Patients with residual tumor are treated with SRS (single dose or hypofractionated) within 3 to 12 months after surgery. Distance between residual tumor and the optic apparatus and therapeutic doses delivered to the tumor were measured and compared with a hypothetical treatment plan that could have been possible early after surgery (MRI at day 1-2).

### Surgical and Radiosurgery Technique

Tumor was accessed through a basal frontotemporal craniotomy. An extradural clinoidectomy was performed in 74% of patients. In the absence of an arachnoid dissection plane between the tumor and the arterial wall of the internal carotid artery/branches, a tinny tumor sheet was left in place without disconnecting tumor off vessel adventitia. No endeavor was made for excision of the CS extension in cases where oculomotor function was intact. Specific consideration was paid to the excision of the tumor constituent positioned inferolateral to the optic nerve and inferomedial to the distal dural ring. In this site, the unresected dura mater was cautiously coagulated while the optic chiasma, vascularizing perforators, and ophthalmic artery were kept under visual control. A fat graft (harvested from infratemporal fossa) is placed between coagulated dural implantation sites, residual tumor, and the optic apparatus. This autograft is thinned out and positioned to protect the optic nerve/anterior chiasm and then fixed in place with Tisseel (Baxter Inc), biological glue.

We performed SRS using Leksell Gamma Knife (Perfexion and ICON, Elekta Instruments, AB), stereotactic imaging (bone computed tomography, MRI multiple sequences). Marginal doses of 12 to 14 Gy are delivered for World Health Organization grade I meningiomas. When the tumor is in contact or is encasing the optic apparatus, we used hfRS of 24 or 25 Gy in 5 fractions on consecutive days.

### Statistical Analysis

Categorical variables were expressed as numbers and percentage. Quantitative variables were expressed as means and range. The Kruskal-Wallis test was used to evaluate the difference between independent measures. Mean fat volume at different time points were compared using ANOVA and the POST HOC Tukey HSD test analysis. The OriginLab was used for the analysis. Statistical significance was defined by *P* values < .05.

## RESULTS

The mean follow-up was 65.57 months (range, 28-128 months). Twenty-three of 71 (32.4%) patients undergoing parasellar meningioma surgery underwent OPN and formed the cohort of this study (see Table 1). GTR was achieved in 6 cases (26%). In the 17 patients with residual tumors (74%), upfront SRS was performed in 14 (82.3%) at various time points after surgery. In 3 patients (17.6%), a radiological follow-up was preferred over SRS because of the small size of the residue. Only 1 patient developed slow asymptomatic progression of residual tumor after SRS which has not yet required further treatment and currently continues clinical and radiological follow-up. Single-fraction SRS was performed in 6 patients (42.8%) at a mean of 5.16 (range, 3.3-10) months after surgery. The mean delivered marginal dose in single fraction was 13 Gy (range 12-14), and the mean maximal dose to optic nerve was 6.72 Gy (range 1.9-8.3). The mean target volume dose was 3.75 cm^3^ (range 0.48-7.55), and the prescription isodose volume was 7.19 cm^3^ (range 0.71-23.7).

**TABLE 1.** Demographic Data: Patients' Characteristics, Their Preoperative and Postoperative Symptoms Are Showed As Well As the Tumors Locations, Surgical Approach, and Management of the Residual Tumors

No. of patients	23
Mean FU (range)	70.3 mo (28-128)
Sex	
Male	5 (22%)
Female	18 (78%)
Mean age in y (range)	57.73 (38-85)
Presenting signs and symptoms	
Visual impairment	20 (87%)
Cranial nerve deficit	2 (8.7%)
Seizure	1 (4.3%)
Headache	1 (4.3%)
Exophthalmos	1 (4.3%)
Tumor location	
Anterior clinoid	13 (57%)
Spheno-orbital	7 (30%)
Tuberculum sellae	3 (13%)
Optic canal invasion	7 (33%)
CS invasion	4 (17.4%)
Surgical approach	
Basal frontotemporal	7 (30.4%)
Basal frontotemporal and clinoidectomy	16 (69.6%)
GTR	6 (26%)
Simpson grade	
II	6
IV	17
WHO grading	
WHO I	23 (100%)
Management of the residual portion	17 (74%)
Gamma Knife	14 (82.4%)
Observation	3 (17.6%)
Recurrence/progression	1 (4.3%)
Postoperative visual status^a^	
Improved	13 (65%)
Stable	7 (35%)
Postoperative complications	
New permanent visual impairment	1 (4.3%)
Vascular complications	2 (8.6%)
Cranial nerve deficit	2 (8.6%)
Neurological motor deficit	1 (4.3%)

CS, cavernous sinus; FU, follow-up; GTR, gross total resection; WHO, World Health Organization.

a In the subgroup of patients with a preoperative visual impairment.

The percentage and range are indicated in brackets.

**FIGURE 1.**
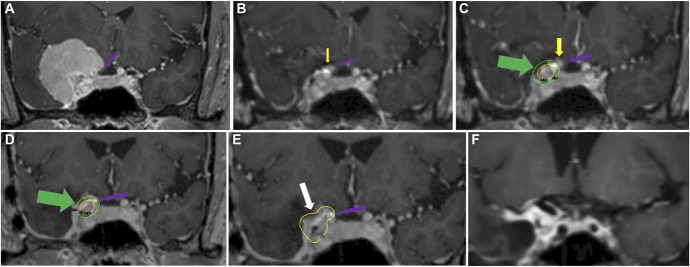
**A**, Preoperative Gd-enhanced T1W coronal MRI image showing a clinoidal meningioma with cavernous sinus invasion and compression of the optic nerve (purple arrow). **B**, Immediate postoperative gadolinium-enhanced T1W MRI showing the fat graft (yellow arrow) between the optic nerve (purple arrow) and the residual tumor. **C**, Hypothetical single-fraction dosimetry with SRS (on immediate postoperative MRI): The green dosimetric curve shows the limit of the 8 Gy dose that passes through the fat graft and stays at a distance from the optic nerve. **D**, MRI at 7 months after surgery (with further fat resorption): The green dosimetric curve of 8 Gy is in contact with the optic nerve (due to fat shrinkage), which did not allow single-fraction treatment. **E**, MRI at 7 months after the surgery showing the yellow dosimetric curve of the tumor and the tumor (white arrow) treated by hfRS. **F**, MRI at 2-year follow-up after hfRS with further shrinkage of the graft and tumor. hfRS, hypofractionated radiosurgery.

Eights patients (57.2%) received hfRS because of the proximity of the optic nerve/chiasma to tumor despite the fat graft. The treatment was performed at a mean of 8.2 (range, 5.1-15.27) months after surgery (Table 2). The difference in delay between surgery and SRS treatment in the 2 groups (single session or hypofractionated) was statistically significant (*P* = .03). In the subgroup of hfRS patients, the radiosurgical plan was compared with a hypothetical treatment plan that could have been made early after surgery (based on immediate postoperative MRI, Figure 1). This showed that the distance between the optic apparatus and the residual tumor immediately after surgery would have been sufficient to deliver a single-session SRS in 75% of the patients in this subgroup, with an upper limit maximum dose to the optic nerve of less than 11.5 Gy (details are summarized in Table 2).

**TABLE 2.** Dosimetric Radiosurgery Data: Are Illustrated Single-Fraction and Hypofractioned Regimens

	Single fraction	Hypofractionated
No. of patients	6 (42.8%)	8 (57.2%)
Mean delay between surgery and GK treatment (range)	5.16 (3.3-10)	8.2 (5.1-15.27)
Mean GK dose to tumors in Gy (range)	13 (12-14)	24.62 (24-25)
Mean GK dose to optic nerve in Gy (range)	6.72 (1.9-8.3)	22.24 (15-25.6)
Mean target volume in cm^3^ (range)	3.75 (0.48-7.55)	5.47 (0.98-13.8)
Mean prescription isodose in volume in cm^3^	7.19 (0.71-23.7)	7.66 (1.23-19.2)
Hypothetical treatment plan (based on immediate postoperative MRI)	12 (85.7%)	2 (14.3%)

GK, Gamma Knife; Gy, gray.

The numbers of patients treated by each modality is indicated.

**FIGURE 2.**
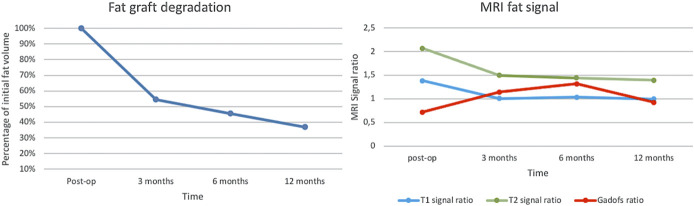
The fat graft volume and its radiological signal over time. The volume is shown in percentage and the radiological signal is detailed in ratio, at the moment of immediate postoperative MRI and at 3, 6, and 12 months after surgery. Approximately 50% of the volume is lost (*P* = .004) and in addition the signal ratio on T1-weighted and T2-weighted images decreases during the first 3 months. The gadolinium-enhanced T1-weighted images increases with time, due to fibroinflammatory response.

**FIGURE 3.**
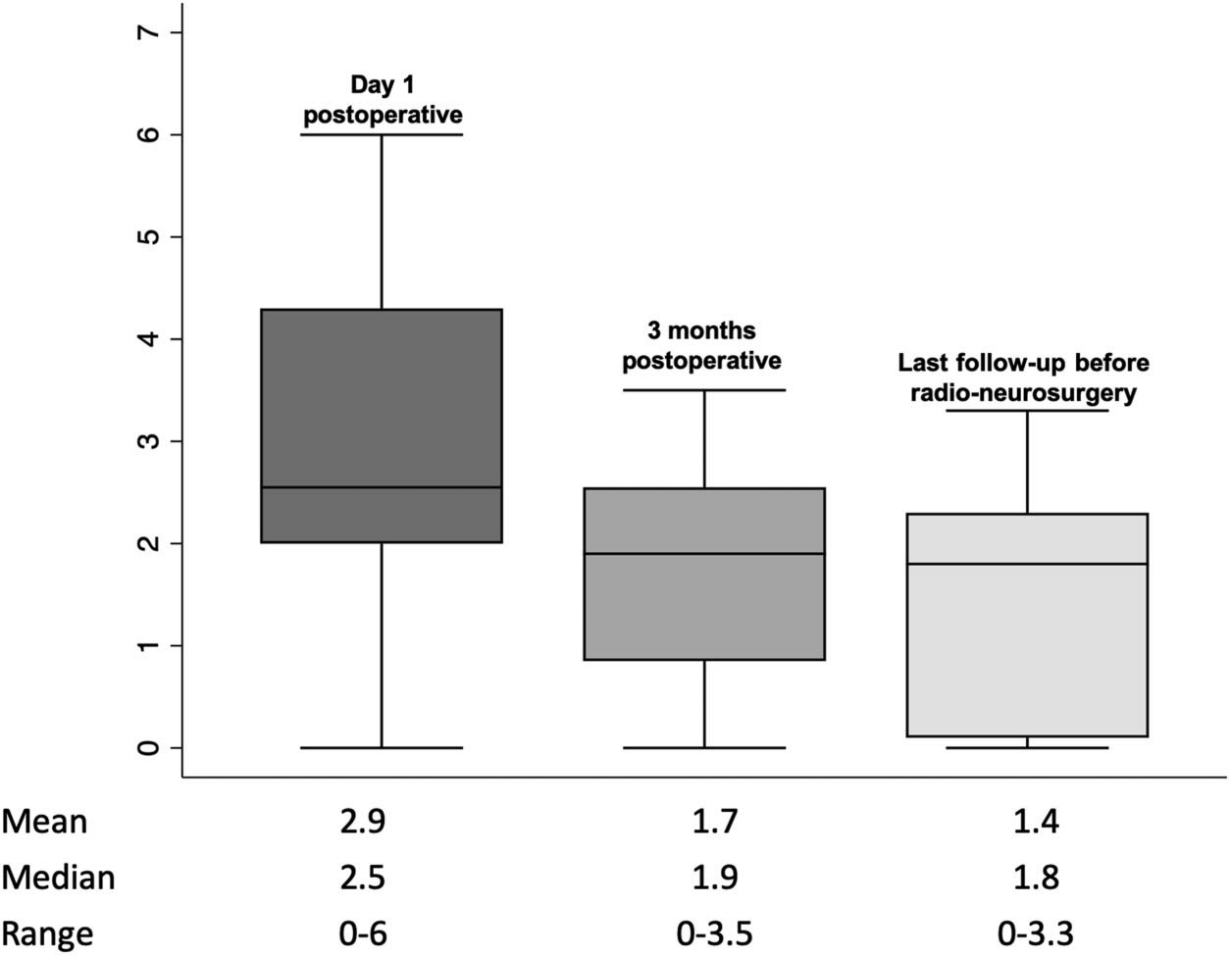
Closest distance in millimeters between the residual tumor and the optic apparatus at day 1 after surgery, 3 months after surgery, and latest follow-up before radiosurgery; the mean, median, and ranges are presented.

Radiological temporal profile of the fat graft showed a progressive loss of volume over time (*P*-value = .03) with a percentage of residual volume, compared with the immediate postoperative control, of 54.5% at 3-month, 45.5% at 6-month, and 37% at 12-month follow-up. No significant difference was found with volume loss in the subanalysis between individual time points (*P*-value = .18 between postoperative time point and 3 months, *P*-value = .86 between 3 months and 1 year) (Table 3). Nevertheless, it is important to note that the curve representing the residual volume shows a steep decline in the first 3 months and a flattening afterward (Figure 2). Sequential analyses of the signal ratio of the fat graft showed a similar decrease over time in the T1-weighted and T2-weighted spin-echo signal intensity, with a steep decline in the first 3 months of 27.2% and 27.6% for T1 and T2 respectively, and a flattening of the curve thereafter (Table 3 and Figure 2). Figure 3 shows the closest distance between the residual tumor and the optic apparatus at different time points. At the same time, GD enhanced T1-weighted spin-echo sequences showed progressive increase in signal intensity between immediate magnetic resonance and 6-month MRI.

**TABLE 3.** Radiological Profile of the Fat Graft: The Radiological Volume and Signal Ratio of the Fat Graft is Detailed

	Immediate postoperative	3-mo FU	6-mo FU	12-mo FU	*P* value
Mean volume in mm^3^ (SD)	1950.2 (1867.3)	1062.5 (1134.9)	888 (973.1)	720.8 (851.2)	*P* = .03
% of the initial volume	100%	54.5%	45.5%	37%	
T1 signal ratio (SD)	1.38 (0.4)	1 (0.4)	1.04 (0.5)	1 (0.4)	*P* = .03
T2 signal ratio (SD)	2.07 (0.6)	1.5 (0.5)	1.44 (0.3)	1.39 (0.4)	*P* = .0003 (T1:2 *P* = .004)
Gadolinium signal ratio (SD)	0.72 (0.6)	1.14 (0.6)	1.32 (0.9)	0.93 (0.5)	*P* = .1

FU, follow-up.

The standard deviation is showed in the bracket.

## DISCUSSION

Although the ideal therapeutic management for meningiomas with symptomatic mass effect is total surgical excision,^9^ GTR is not always feasible at the cranial base,^10^ particularly for lesions invading the CS.^1,11,12^ Most series on meningiomas involving the CS report a recurrence rate of about 60% with subtotal resection compared with a rate of about 5% to 10% in cases of GTR.^1,13^ However, GTR of tumors invading the CS is associated with a high rate (at least 33%) of postoperative neurological deficits and mortality.^13-16^ Moreover, series that report resection of the intracavernous portion of the lesion^17-19^ show GTR rates (42%-58.9%) and tumor recurrence rates (5%-10%)^14,18-20^ similar to those reported in series adopting a conservative strategy (GTR 64.2%; tumor recurrence 8.9%, 95% CI, 6%-11.8%).^1,12^ Recent recommendations advocate for a combined treatment, relying on planned subtotal resection (without intracavernous dissection and tumor removal), followed by SRS on the intracavernous residual tumor.^13,21,22^

In our series of parasellar meningiomas, we identified 23 patients (32%) in whom we performed OPN. These patients had extension to the CS or around carotid artery with unresectable dural attachments. In our series, 14 of 17 patients with residual tumor underwent SRS (82.4%; 42.8% single and 57.2% hfRS). Only 1 patient developed slow progression after SRS. There were no long-term deficits related to the radiosurgical treatment. Proximity of a remnant to the optic apparatus or the pituitary gland can limit the therapeutic physical dose prescribed by SRS to lower the risk of RION or pituitary insufficiency.^23^ According to Quantec,^6^ risk of optic pathways toxicity increases markedly at more than 60 Gy (1.8 Gy/fraction) and more than 12 Gy for single-fraction. Recent studies suggest that safe maximal dose received by the optic apparatus (single fraction) might be as high as 12 Gy.^24^ hfRS can be used when there is close contact or even encasement of the optic apparatus.^25^ Fariselli et al^25^ used Cyberknife (Accuray) for hfRS and reported high local control rates (93% at 5 years) with limited RION (5.1%). A recent systematic review^22^ showed similar progression-free survival at 5 years, with single-fraction SRS inducing more tumor volume regression than fractionated radiotherapy.^22^ Conti et al^26^ described multisession SRS with progression-free survival (PFS) at 2, 5, and 10 years of 95%, 90%, and 80.8%, respectively. Conti et al^27,28^ matched normofractionated stereotactic radiotherapy with CyberKnife-based hypofractionated with no important changes in LC rates. Combs et al^29^ assessed durable results of high-precision photon radiotherapy and established that PFS was 91% at 10 years.^29^ Combs et al^30^ evaluated high-precision radiation therapy RT, with a PFS of 98% at 1 year, 94% at 3 years, 92% at 5 years, and 86% at 10 years.^30^

In most cases, residual tumor left within the CS is anatomically close to the optic nerve cisternal segment. This part of the nerve is often displaced by the tumor within the cistern but tends to move back to its normal position with time, thereby losing the distance obtained at surgery between the nerve and residual tumor. OPN maintaining this distance gained at surgery and facilitate future SRS.^3^

Fat graft placement is not a current gold standard in part because of its inherent nature to shrink over time.^31,32^ Our study shows an early shrinkage with the highest decrease in fat volume occurring during the first 3 months after surgery. This process continues at a slower rate during subsequent follow-up, with more than 60% of fat resorbed at 1 year. Considering these data and the significant difference in mean delay between surgery and SRS treatment in the single-session and hfRS groups (5.16 vs 8.2 months), we then showed that in 75% of the patients treated with hfRS, distance between the optic apparatus and residual tumor immediately after surgery would have been sufficient to deliver single fraction. Hence, the space produced by the graft, the possibility of its early shrinkage, and known tumor remaining volume outlined on postoperative MRI deserve the use of early postoperative SRS when anatomic and dosimetry conditions are optimum. The basis to delay systematic SRS, to avoid scarring and amplified surgical dangers in case of reoperation,^33^ should be balanced against the benefit of excellent SRS tumor control results.^2,22,34^

Few series suggest that hypofractionated SRS would be more appropriate in patients with close proximity to the optic apparatus. Yet, the advantages of such an approach over single fraction (whenever feasible) still need to be proved in terms of local control and radiation-induced toxicity.^35^ Radiobiological modeling^36^ favors the use of single-fraction SRS rather than fractionation for tumors with low alpha/beta ratio (late responding tissues), including meningiomas.^37,38^ The use of the fat graft has brought up new challenges concerning radiological postoperative image interpretation, particularly concerning evaluation of residual tumor and its relationship to the optic apparatus.^32,39^ The significant decrease of fat signal ratio at 3-month MRI limits the delineation of the fat graft and optic nerve after this time point. There was also an increase of fat signal ratio (fat vs pons) on T1-weighted images after gadolinium injection. This is likely explained by graft progressive fibroinflammatory involution, leading to more prominent hypointensity and an increase in contrast enhancement. Therefore, tumor delineation from the fat graft becomes difficult 3 months after surgery, similar to earlier studies.^32,40-42^ The pathogenesis in-back-of fat graft contrast enhancement is probably explained by an adaptation to the receiver background with boosted vascularization required for its survival.^32,43,44^ This fact must be kept in mind as the fibrosis could be misjudged as recurring tumor. A cautious examination of the preoperative images is essential to identify the original tumor and then to categorize the autograft fat in early postoperative images. Based on this study, we have changed our postoperative policy to perform an MRI after 6 weeks to enable the possibility of adjuvant SRS within the first 3 months.

### Limitations

Our study has several inherent limitations. The first is directly related to the retrospective nature, with all bias derived from this type of study design. The second is the limited number of patients. Such findings should be validated in larger cohorts. A third limitation is related to the follow-up period after surgery. Although we followed the patients every 3 months, the exact time after surgery of fat degradation remains difficult to establish precisely.

## CONCLUSION

OPN is safe and useful after resection of parasellar meningiomas. For residual tumors that will require adjuvant SRS, OPN allows delivering single-fraction SRS in most patients, provided this is performed within the first 3 months after surgery. Anatomic delineation used during SRS with respect to differentiation of fat graft from optic apparatus and the residual tumor is easier within the first 3 months after surgery.

## COMMENTS

This is a nice paper describing the evolution in terms of the size and signal characteristics of the free fat graft placed at surgery, to separate the optic nerve and chiasm from the residual tumor. Based on this report, the authors conclude that the best time to consider single fraction SRS is within less than 3 months after surgery. Within this time frame, the tumor can be seen separate from the tumor on MRI sequences. Beyond this window, the best option for radiotherapy would be hypofractionated SRS since the fat regresses in volume and becomes indistinguishable from the tumor. As far as the management strategy for the residual tumor is concerned, whether radiosurgery vs hfSRS vs observation, is not addressed in this paper.

Chandranath Sen

*New York, New York, USA*
